# Bicycle helmet laws and persistent racial and ethnic helmet use disparities among urban high school students: a repeated cross-sectional analysis

**DOI:** 10.1186/s40621-016-0086-3

**Published:** 2016-09-05

**Authors:** John D. Kraemer

**Affiliations:** Department of Health Systems Administration and O’Neill Institute for National & Global Health Law, Georgetown University, 3700 Reservoir Road, NW, 231 St Mary’s Hall, Washington, DC 20057 USA

## Abstract

**Background:**

Bicycle helmet laws generally increase helmet usage, but few studies assess whether helmet laws reduce disparities. The objective of this study is to assess changes in racial/ethnic disparities in helmet use among high school students in urban jurisdictions where laws were previously determined to increase overall helmet use.

**Methods:**

Log-binomial models were fit to four districts’ 1991–2013 Youth Risk Behavior Survey (YRBS) data. Post-regression predictive margins were used to calculate adjusted bicycle helmet use proportions, assess before-to-after changes in race/ethnicity specific helmet use, and estimate changes in disparities from jurisdictions’ white subpopulations.

**Results:**

Helmet use among white students increased by 10.2 percentage points in two Florida counties (*p* < 0.001), 20.1 points in Dallas (*p* < 0.001), and 24.4 points in San Diego (*p* < 0.001). Increases among African Americans were 6.1 percentage points in the Florida counties (*p* < 0.001), 8.2 points in Dallas (*p* < 0.001), and 6.3 points in San Diego (*p* = 0.070). Use increased among Latino students in the Florida counties (4.3 percentage points, *p* = 0.016) and Dallas (6.2, *p* = 0.002), but not significantly in San Diego. San Diego helmet use among Asian students increased by 12.8 percentage points (*p* < 0.001). Because helmet use increased more for white students, helmet laws were associated with increased disparities. In the Florida counties, disparities increased significantly by 5.9 percentage points for Latino students (*p* = 0.045). San Diego disparities worsened by 18.1 (*p* < 0.001), 21.3 (*p* < 0.001), and 11.6 (*p* = 0.013) percentage points among African American, Latino, and Asian students respectively. Dallas disparities increased by 11.9 (*p* = 0.015) and 14.0 (*p* = 0.003) percentage points among African American and Latino students. Increased disparities generally persisted for follow-up time of at least a decade. Main study limitations include the possibility of helmet use reporting error and limited socioeconomic variables in YRBS datasets.

**Conclusions:**

Helmet use increased across racial/ethnic subpopulations, but greater increases among white students increased disparities. Policymakers should couple laws with other approaches to reduce helmet disparities and cycling injuries.

**Electronic supplementary material:**

The online version of this article (doi:10.1186/s40621-016-0086-3) contains supplementary material, which is available to authorized users.

## Background

Bicycle helmets strongly protect against head and facial injuries when cyclists are involved in crashes (Thompson et al. 2000), but U.S. adolescent helmet use remains low and significant racial and ethnic disparities in adolescent helmet use exist. In the most recent national high school student survey, white youth were twice as likely to wear helmets “sometimes” or more frequently than African American or Latino students (Kann et al. 2014). Similarly, a three-city study of 5^th^ graders in Birmingham, Alabama; Houston, Texas; and Los Angeles County, California, found that white students were over 30 percentage points more likely to wear helmets than their African American or Latino counterparts. Racial/ethnic disparities remained but were halved when adjusted for household income (Schuster et al. 2012).

Most studies find that mandatory helmet laws increase helmet use (Macpherson & Spinks 2008; Karkhaneh et al. 2006). Whether laws reduce helmet disparities is, however, uncertain. From a theoretical perspective, helmet laws inducing a high degree of compliance would be expected to reduce disparities as helmet use across subpopulations converge near universality. Achieving such rates would likely require cyclists to believe enforcement is common and penalties are meaningfully large (Polinsky & Shavell 2007; Jennings & Mieczkowski 2011). Conversely, if helmet laws serve primarily an informational or norm-setting function, effects may vary across subgroups proportional to the extent to which laws change perceived benefits of helmet use or behavioral norms. Subgroup differences in both baseline levels and amenability to changes may moderate helmet laws’ effectiveness (Burris & Wagenaar 2013). Alternatively, laws may engender smaller effects among more marginalized populations even if there is no difference in expected benefits if costs of compliance are proportionally greater (National Highway Traffic Safety Administration 2004).

Empirical evidence is mixed, and helmet laws are associated with greater, lesser, and unchanged disparities in different studies. A direct observation study in New York’s Queens borough identified greater helmet use increases among white than African American children following a helmet law and educational program (Abularrage et al. 1997). Similarly, a small study in Georgia found significant helmet use increases across grade-school subpopulations, but post-law helmet increases were greatest among white females (Gilchrist et al. 2000). Another study found knowledge of the Georgia law to be higher among white than nonwhite respondents (Schieber et al. 1996). On the other hand, several studies find comparable effects across subpopulations. A large cross-sectional study found that helmet use was approximately 50 percentage points higher in Florida counties with helmet laws than those without, and the difference was consistent across white, African American and “other” grade-school students (Kanny et al. 2001). This accords with a Canadian study finding that helmet use increased by approximately the same amount in higher and lower-income neighborhoods from 2 years before to two years after Alberta’s law (Hagel et al. 2006). Finally, an Oregon survey-based study found that helmet ownership increased more among low-income than high-income students following the state’s law, but it did not assess race/ethnicity (Ni et al. 1997).

To date, no American studies have assessed long-term effects on helmet disparities, but several Canadian studies reached intriguing results. A study in Alberta found roughly comparable increases in helmet use across neighborhood income levels for children under age 13 four years after the provinces’ law, but, among 13–17 year-olds, significant increases only occurred in higher-income neighborhoods (Karkhaneh et al. 2011). Another study found that Ontario’s helmet law had greater effects in low and middle-income areas than high-income parts of Toronto, significantly reducing disparities across income-level regions of the city (Parkin et al. 2003). However, the effect was short-lived; by six years after the law, helmet use in low and middle-income areas had reverted to baseline levels and disparities were reestablished (Macpherson et al. 2006). These findings are consistent with two non-legislative educational and helmet distribution interventions in Ontario and Quebec that found greater effects among youth in higher-income than lower-income areas (Parkin et al. 1993; Farley et al. 1996). None of these studies assessed racial/ethnic disparities.

This study extends an earlier analysis that found helmet laws increased bicycle helmet use among high school students in four urban public school districts using a variety of strategies for causal inference (Kraemer 2016). This study uses the same dataset to understand two questions: 1. were bicycle helmet laws associated with changes in racial and ethnic helmet disparities and 2. if changes were detected, did they persist over the long run or revert?

## Methods

### Setting and participants

This study uses school district-level Youth Risk Behavior Survey (YRBS) data, the collection of which are supported by the Centers for Disease Control and Prevention (CDC) for a select set of large, urban districts. District-level data enable crisp ascertainment of exposure to helmet laws, whereas state YRBS datasets do not identify municipalities, so respondents not subject to a state law may be inaccurately classified if they are still subject to a municipal law. YRBS methods are fully described elsewhere (Kann et al. 2014; Centers for Disease Control and Prevention (CDC) et al. 2013). Briefly, though, YRBS data are collected biennially via a two-stage cluster sampling approach, with inverse probability weights applied to adjust for oversampling and non-response. Jurisdictions use a standard questionnaire with optional additions or deletions.

Districts were selected as previously described (Kraemer 2016), with included jurisdictions being those that had data meeting CDC’s standards to be population-representative, a state or municipal helmet law, and helmet use data from the surveys immediately before and after their law. Four districts met these criteria: Dallas, Texas; San Diego, California; and Broward and Miami-Dade Counties, Florida. Weighted survey data was available from 1991 through 2013 for all jurisdictions except Dallas (1991–2011) (Kraemer 2016).

The jurisdictions' helmet laws have been summarized previously (Kraemer 2016). Briefly, California’s state law requires helmet use by <18-year-old cyclists, with the mandate taking effect on January 1, 1994, and phasing in a $25 penalty one year later. A Dallas all-age municipal ordinance, with graduated fines ranging from $10 to $25, went into effect on September 1, 1996. (It was amended to <18 only in 2014.) A Florida <16-year-old law went into effect on January 1, 1997, with its $15 penalty phased in on January 1, 1998 (Kraemer 2016). While there exists limited data on enforcement, it appears unvigorous (National Highway Traffic Safety Administration 2004; Borglund et al. 1999).

Respondents were included if they were within the relevant jurisdiction’s targeted age group (all ages in Dallas, <18 in San Diego, and <16 in the Florida counties) and had ridden bicycles within the last year. Racial/ethnic subpopulations with consistently fewer than 50 respondents per survey were excluded from analyses because small samples precluded stable estimates. For Dallas and the Florida counties, analyses were limited to the white, non-Latino; African American, non-Latino; and Latino groups. San Diego analyses also included the Asian subpopulation.

### Approach for causal inference

Ideally, comparison to concurrent controls in a jurisdiction without bicycle helmet laws would be used to assess whether and to what extent helmet laws impacted race/ethnicity-specific helmet use rates and racial/ethnic disparities. Unfortunately, the only urban school district with helmet data for the appropriate years, Chicago, has too small of a sample to permit subgroup controls. Instead, the primary analysis in this paper relies on the general causal inference that laws increased helmet use in these jurisdictions, as previously found (Kraemer 2016). Thus, it first assesses before-to-after changes within each subpopulation to estimate the potential subgroup-specific effect. Second, it assesses changes in disparities as the difference in subgroup differences from before-to-after each law’s implementation.

### Variables

YRBS variables are standardized and fully described elsewhere (Centers for Disease Control and Prevention 2014). The principal outcome variable, helmet use, was dichotomized (sometimes, most of the time or always vs. rarely or never) from a five-point Likert scale, which is consistent with CDC’s standard approach (Kann et al. 2014). Surveys conducted prior to each jurisdiction’s law’s effective date were coded as “before,” and surveys conducted one year or more after the effective date were coded as “after.” The helmet use question has a 1-year recall period, so surveys conducted within one year of the law’s effective date were excluded from the main analyses to prevent exposure misclassification. In Dallas and the Florida counties, the “before law” period ended with 1995 and the “after law” period began in 1999. For San Diego, survey years aligned with the California law’s phased implementation. Thus, the 1993 survey was coded as “before,” the 1995 survey corresponded to the period during which helmets were mandated without a penalty, and 1997 was the first mandate-and-penalty survey (Kraemer 2016). Race/ethnicity was based on respondent self-report from provided categories (American Indian/Alaska Native, Asian, Black/African American, Hispanic/Latino, White), “other,” and multiple choices could be selected from 1999 onward. In all jurisdictions, the number of respondents identifying as American Indian/Alaska Native, other, or multiple races were too small for inclusion.

Over time, the sample size of respondents who met inclusion criteria fell in most jurisdictions, particularly in Dallas and San Diego. This resulted principally from reductions in the intended number of sampled students, though there were also long-term secular trends toward fewer respondents riding bicycles, which most came into play in late 1990s and 2000s (Kann et al. 2014; Kann et al. 1995; Kann et al. 2000; Eaton et al. 2006). Based on a prior analysis (Kraemer 2016), survey years were combined to maintain stable estimates if either the total sample was below 800 or any included subpopulation was at or below 100. The following years were merged into combined time periods. For Dallas: 2001 and 2003, 2005 and 2007, and 2009 and 2011. For Miami-Dade and Broward Counties: 2007 and 2009. For San Diego: 2003 and 2005, 2007 and 2009, and 2011 and 2013. Additionally, 1991 was dropped because its sample included fewer than 100 respondents in all non-white groups and merging it with 1993, the last pre-law survey, would potentially bias the pre-law baseline.

Age and sex were included as control variables because they were associated with helmet use in previous studies and might be correlated with race/ethnicity among cyclists (Kann et al. 2014; Kanny et al. 2001; Kraemer 2016). Age was categorized as 14 years and below, single year categories from 15 to 17, and 18 and older.

### Statistical methods

#### Models

Analyses compared age-and-sex-adjusted, race/ethnicity-specific proportions of helmet use over time. Adjusted proportions were calculated in two steps. In the first, generalized linear models with log link functions and binomial error distributions (log-binomial models) were fit. Each model regressed helmet use on indicator variables for time period, race/ethnicity, and their interaction. Indicator variables were also included for sex and age. Separate models were fit for Dallas, San Diego, and the combined Florida counties, which were found to be comparable in a prior analysis (Kraemer 2016). Models were restricted to respondents who reported riding a bicycle in the last year and were within the law’s targeted age in that jurisdiction.

After regression models were fit, adjusted proportions of helmet use in each subpopulation for each time period were calculated using predictive margins with covariates held at their observed values. Within each racial/ethnic group, the adjusted before-to-after difference in helmet use was tested by performing contrasts of predicted values. Adjusted before-to-after changes in disparities were calculated by performing contrasts on the difference in before-to-after differences in helmet use between the white subpopulation and each other group (Williams 2012). The main analyses compared the first post-law year to the last pre-law year, but before-after differences and changes in disparities for all periods were calculated and graphed to visualize long-term trends.

#### Incorporation of complex sampling

Surveys were merged with strata kept unique between years and districts to preserve the sampling structure. Taylor series linearization was used to adjust standard errors for the sampling structure, and inverse probability weights were applied to generate population-representative estimates. As previously described (Kraemer 2016), some sampling variables had to be reconstructed for the 1991, 1993, and 1997 Dallas datasets, which does not affect point estimates but might introduce minor error into those years’ confidence intervals. (See the methods appendix to Kraemer 2016 for further explanation.) Statistical analyses used Stata 14.1. Analysis code is provided in Additional file 1: Analysis Code.

#### Ethics

CDC’s institutional review board oversees YRBS protocols. Students may opt out, and school districts follow local parental permission procedures (Kann et al. 2014; Centers for Disease Control and Prevention (CDC) et al. 2013). Public-use datasets are stripped of potential identifiers before being released. Georgetown University’s IRB does not require additional oversight of the study because it used anonymized, publicly available data.

## Results

The full dataset included 85,998 respondents across 46 surveys. A total of 35,255 met inclusion criteria of being within their jurisdiction’s targeted age group and being a bicycle rider. A total of 2609 respondents were excluded because members of racial/ethnic subpopulations that were too small for stable estimates, and 396 when San Diego’s 1991 survey was excluded, for a final sample of 32,250. Respondent characteristics are provided in Table 1.

**Table 1:** Characteristics of included respondents subject to each jurisdiction’s helmet law

	Dallas	San Diego	Miami-Dade & Broward Counties
	% (CI)	% (CI)	% (CI)
Characteristics			
Female sex, % (CI)	44.4 (43.2, 45.7)	41.9 (40.6, 43.2)	46.3 (45.1, 47.6)
Race/Ethnicity, % (CI)			
White, non-Latino	10.8 (9.8, 12.0)	33.7 (32.2, 35.2)	23.6 (22.0, 25.3)
African American, non-Latino	40.6 (38.8, 42.4)	13.9 (12.9, 14.9)	28.7 (26.8, 30.7)
Latino	48.6 (46.8, 50.4)	37.9 (36.4, 39.4)	47.7 (45.7, 49.7)
Asian	---	14.5 (13.6, 15.4)	---
Age, % (CI)			
14 and under	12.3 (10.8, 14.0)	18.9 (17.1, 20.7)	27.6 (26.3, 29.0)
15 years old	31.7 (29.6, 34.0)	30.7 (28.9, 32.4)	72.4 (71.0, 73.7)
16 years old	26.6 (24.5, 28.8)	28.1 (26.4, 29.9)	---
17 years old	18.5 (16.7, 20.5)	22.4 (20.5, 24.4)	---
18 and over	10.9 (9.4, 12.5)	---	---
Sample Sizes, n	11,514	9871	10,865
1991	1894	---	961
1993	1847	1047	809
1995	1776	1072	849
1997	875	1309	935
1999	934	898	987
2001	951	873	875
2003	967	798	948
2005	631	743	963
2007	561	743	686
2009	466	886	867
2011	612	823	1088
2013	---	679	897

Sample sizes (n) are unweighted. Percentages and their confidence intervals for respondent characteristics are weighted. “---” denotes that that subpopulation or survey year was excluded from the analysis for reasons described in the methods

**Fig. 1:**
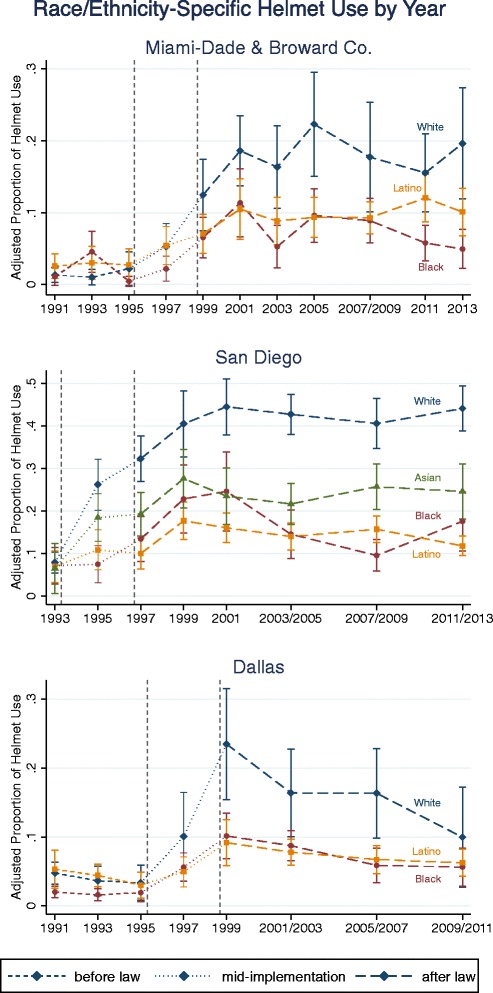
Time series of race/ethnicity-specific helmet use in each jurisdiction. Vertical reference lines correspond to the end of the pre-law period and start of the post-law period. Error bars represent 95 % confidence intervals

Across jurisdictions and subpopulations, helmet use increased when helmet laws entered effect (Table 2 and Fig. 1). In Dallas, confounder-adjusted helmet use increased significantly by 6.2 percentage points (95 % CI 2.3–10.0) among Latinos, 8.2 (95 % CI 4.7–11.7) among African Americans, and 20.1 (95 % CI 11.7–28.5) among whites. San Diego had significant increases in helmet use among Asian (12.8 percentage points, 95 % CI 5.0–20.5) and white (24.4 percentage points, 95 % CI 18.5–30.3) cyclists. There was a marginally significant 6.3 percentage point (95 % CI −0.50–13.0) increase among African Americans and a non-significant 3.1 percentage point (95 % CI −2.1–8.3) increase among Latinos. In the Florida counties, significant increases of 4.3 (95 % CI 0.8–7.9), 6.1 (95 % CI 3.2–9.0), and 10.2 (95 % CI 4.8–15.7) percentage points were observed among the Latino, African American, and white subpopulations, respectively. (Unadjusted results are comparable; see Additional file 2: Table S1.) At the last time period, helmet use remained significantly above pre-law levels in all groups except for the white subpopulation in Dallas, where there remained a marginally significant 6.6 percentage point (95 % CI −1.1–14.3) increase (Additional file 3: Figure S1 and Additional file 2: Table S2).

**Table 2:** Adjusted increases in helmet use and disparities from before-to-after helmet law implementation

	Dallas		San Diego				Miami Dade & Broward Counties	
	Mandate & Penalty		Mandate Only		Mandate & Penalty		Mandate & Penalty	
	Δ% (CI)	p	Δ% (CI)	p	Δ% (CI)	p	Δ% (CI)	p
Before-to-After Increase in Helmet Use								
White, Non-Latino	20.1 (11.7, 28.5)	<0.001	18.3 (11.9, 24.8)	<0.001	24.4 (18.5, 30.3)	<0.001	10.2 (4.8, 15.7)	<0.001
African American, Non-Latino	8.2 (4.7, 11.7)	<0.001	0.3 (−5.8, 6.5)	0.915	6.3 (−0.5, 13.0)	0.070	6.1 (3.2, 9.0)	<0.001
Latino	6.2 (2.3, 10.0)	0.002	4.0 (−2.0, 9.9)	0.188	3.1 (−2.1, 8.3)	0.242	4.3 (0.8, 7.9)	0.016
Asian	---	---	11.9 (3.8, 20.0)	0.004	12.8 (5.0, 20.5)	0.001	---	---
Increase in Disparity								
White, Non-Latino	Ref.	Ref.	Ref.	Ref.	Ref.	Ref.	Ref.	Ref.
African American, Non-Latino	11.9 (2.3, 21.4)	0.015	18.0 (8.8, 27.3)	<0.001	18.1 (9.3, 27.0)	<0.001	4.2 (−1.8, 10.1	0.169
Latino	14.0 (4.7, 23.2)	0.003	14.4 (5.8, 22.9)	0.001	21.3 (13.0, 29.6)	<0.001	5.9 (0.1, 11.7)	0.045
Asian	---	---	6.4 (3.9, 16.7)	0.221	11.6 (2.5, 20.8)	0.013	---	---

“---” denotes that the relevant subpopulation was not included because it was too small for stable estimates

**Fig. 2:**
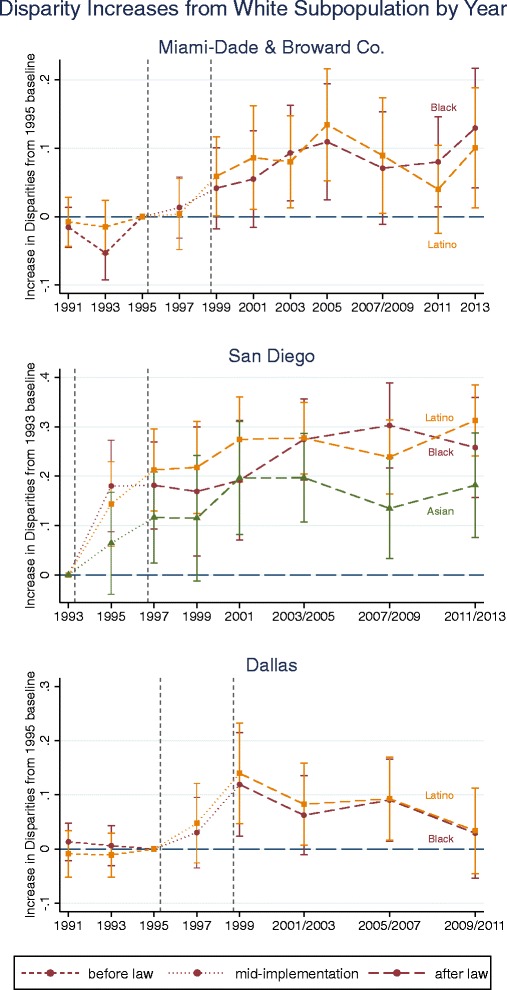
Change in helmet use disparity compared to each jurisdiction’s white subpopulation. Comparisons are from the pre-law baseline. Horizontal reference line corresponds to no change in disparity. Vertical reference lines correspond to the end of the pre-law period and start of the post-law period. Error bars represent 95 % confidence intervals

Because helmet use increased most in all jurisdictions’ white subpopulations, disparities increased (Table 2 and Fig. 2). In Dallas, the disparity compared to the white subpopulation increased significantly by 11.9 percentage points (95 % CI 2.3–21.4) among African American students and 14.0 percentage points (95 % CI 4.7–23.2) among Latinos after the law’s implementation. In San Diego, disparities worsened significantly by 11.6 (95 % CI 2.5–20.8), 21.3 (95 % CI 13.0–29.6), and 18.1 (95 % CI 9.3–27.0) percentage points, respectively, among the Asian, Latino, and African American subpopulations. In the Florida counties, the disparity worsened significantly among Latino students by 5.9 percentage points (95 % CI 0.1–11.7), but the change was not significant among African American students (4.2 percentage points, 95 % CI −1.8–10.1). (Unadjusted results are comparable and presented in Additional file 2: Table S1.) Increased disparities retained their approximate magnitude throughout the study period in San Diego and the Florida counties (see Fig. 2, Additional file 2: Table S3, and Additional file 3: Figure S2). In Dallas, the increase in disparities shrank and became statistically insignificant from the pre-law period for both African Americans (*p* = 0.492) and Latinos (*p* = 0.403) by the last time period. This resulted from reduced helmet use in the white group and not increases among African American or Latino students.

## Discussion

Broadly, this paper makes two findings. First, helmet use increased across jurisdictions and racial/ethnic subpopulations after laws went into effect. While in some instances, helmet use fell from its post-law peak (as with white students in Dallas), virtually every subpopulation continued to wear helmets at a rate exceeding its pre-law baseline. Second, because helmet use increased most among each jurisdiction’s white subpopulation, helmet use disparities increased after laws went into effect and persisted. The only exception was Dallas, where the increase in disparities closed and became non-significant because, while helmet use declined from its post-law peak in all subpopulations, the white group’s decline was greatest.

There are several reasons to believe these findings are causal. First, a prior analysis used multiple strategies to infer the helmet laws’ causal effect in each jurisdiction. While that study used approaches that were not available at the subpopulation level, it is reasonable to infer that, absent other explanations, causal effects at the population level apply to subpopulations. Second, similar before-to-after changes in helmet use and disparities were observed across jurisdictions. This consistency makes it less likely that associations were due to confounders arising coincident with the helmet law in each jurisdiction.

### Limitations

Standard YRBS limitations apply. In particular, data are self-reported and therefore incorrect reporting is possible (Centers for Disease Control and Prevention (CDC) et al. 2013). While validity cannot be directly evaluated, two assessments of helmet use test-retest reliability found kappa scores of 0.76 (for wore helmet “never” or “rarely”) (Brener et al. 2002) and 0.50 (for “always” wore helmet) (Brener et al. 1995). The latter score was likely reduced because observed prevalence was low (0.4 %), which increases expected agreement and decreases kappa. Imperfect helmet use reliability should bias toward the null.

While helmet use can be reliably measured via other techniques, such as direct observation, ascertaining and assigning race and ethnicity by observation raises both normative and measurement issues (Smith 2001; Kraemer et al. 2012; Kraemer et al. 2015). While self-reported race and ethnicity is still subject to recording mistakes, it avoids external assignment and thus is more likely to represent the social constructs to which race and ethnicity relate and which affect helmet use. The race categories used in YRBS are coarse, which precludes capturing important nuance about disparities (for example, “Asian” includes people of both East and South Asian heritage), though finer categories would cause tradeoffs with statistical power (Ulmer et al. 2009).

YRBS contains few social and demographic variables. As a result, it is possible that unobserved variables confound the associations identified in this study. To confound results, absent variables would have to vary within cities over time, cause differences in helmet use, and correlate with race/ethnicity. The most conspicuously absent variable is wealth, which has changed in complex relationships with race and ethnicity in large U.S. cities over the last 25 years, with demographic shifts generally accelerating in the 2000s (Frey 2015).

Out-of-jurisdiction controls were not available due to no control jurisdiction having sufficient sample size for race or ethnicity-specific comparison groups. As a result, causal inferences should be made cautiously. However, as discussed above, inferring causality is reasonable in light of prior evidence that helmet laws in the same jurisdictions causally increased helmet use.

As is often the case with legal interventions, it is not possible to disentangle the effect of helmet laws from accompanying efforts, such as educational campaigns. With the exception of a nurse-led educational outreach program in Broward County elementary schools (Borglund et al. 1999), there is little documentation of educational or sensitization programs in the studied jurisdictions. As a result, this paper’s findings are more likely to represent average implementation than “ideal” implementation.

Finally, students at large, urban school districts are likely not to be representative of US high school students as a whole (Centers for Disease Control and Prevention (CDC) et al. 2013). However, the range of jurisdictions represented in this study are likely representative of large, urban districts, so it is reasonable to generalize this paper’s findings to students of such school districts.

### Policy considerations

Policymakers generally have a utilitarian duty to improve public health and an egalitarian duty to reduce disparities (Powers & Faden 2006). In this study, helmet laws appear to increase aggregate helmet use but also worsen disparities, creating an ethical tension. Had the laws increased disparities by worsening outcomes among marginalized groups, distributive justice might require their repeal or non-enactment if other ameliorative approaches did not succeed (Rawls 1971). However, repeal would not benefit marginalized groups in the case where—as here—laws improve outcomes across subpopulations but by unequal amounts.

Instead, in this case, policymakers should take at least two steps to mitigate disparities.

First, it is important to better understand and address why helmet laws have disparate impact, and this is a topic for further research. Ameliorative strategies would be different if, for example, income levels mediate the ability to comply with helmet laws and are therefore the principal root cause of laws’ differential effects than if helmet laws were less effective in some populations because risk communications that accompanied laws were poorly targeted across groups. Causes are likely at least partially locality-specific.

Second, health officials should take steps to reduce injury risk independent of helmet laws. Several studies have previously found that infrastructure for vulnerable road users—including for cyclists—tends to be more dangerous in areas where more minority and lower-income residents reside (Chaney & Kim 2013; Silverman et al. 2013; Chakravarthy et al. 2012). Aside from helmet use, improving the urban built environment to reduce disparities in the safety of cycling infrastructure could make overall injury risks more just (Mulvaney et al. 2015). Specific to helmets, multifaceted helmet promotion programs aimed at populations with lower use should be employed (White et al. 2009). While these should be context-specific, they may include culturally competent educational campaigns, helmet distribution, and efforts to reframe helmet norms (Owen et al. 2011; O’Callaghan & Nausbaum 2006).

Additionally, policymakers should consider whether to increase helmet law enforcement, which is usually minimal. On one hand, greater enforcement would likely lead to increased helmet use and could reduce disparities if it led to high use across subpopulations (Gilchrist et al. 2000). On the other hand, greater enforcement would result in groups with lower helmet use receiving more citations and might further burden marginalized groups. There exists evidence that bicycle laws, including helmet laws, are enforced pretextually against members of minority racial and ethnic groups in some cities (National Highway Traffic Safety Administration 2004; Benning 2014; Stanley & Zayas 2015; Goodyear 2015). Whether greater enforcement would reduce this (by reducing police discretion) or increase it (by increasing incentives to issue citations) is uncertain.

## Conclusion

On balance, bicycle helmet laws are sound policy and increase helmet use (Macpherson & Spinks 2008). Importantly, in this study, helmet use was observed to increase across racial/ethnic subpopulations. However, the greater increases observed among white than minority populations should cause policy makers to redouble efforts to increase helmet use and reduce injury risks among minority populations.

## Additional files

Additional file 1: Appendix C: Analysis Code. (RTF 10 kb) Additional file 2: Appendix A: Supplemental Tables. (DOCX 100 kb) Additional file 3: Appendix B: Supplemental Figures. (DOC 333 kb)

## Abbreviations

CDC: U.S. Centers for Disease Control and Prevention
YRBS: Youth Risk Behavior Survey
