# Supplementary material for: Bicycle helmet laws and persistent racial and ethnic helmet use disparities among urban high school students: a repeated cross-sectional analysis
Source: Inj Epidemiol. 2016 Sep 5;3(1):21. doi: 10.1186/s40621-016-0086-3 (PMC5011068; doi:10.1186/s40621-016-0086-3)
Supplement: Additional file 1: — Appendix C: Analysis Code. (RTF 10 kb) [file 40621_2016_86_MOESM1_ESM.rtf]

Appendix C: Analysis Code/* Instructions for use: Copy and paste text into Stata do file to run.    		For questions, data cleaning code, etc, contact the paper's author.		Data sets are publicly available from the CDC, though Dallas datasets require separate permission from Dallas’ YRBS office.   */		*=====================================================================**	TABLE 1: RESPONDENT CHARACTERISTICS								  |*=====================================================================*	//A. Dallas	tab rode_bicycle if sitecode=="DA" & target_dallas==1 & race_disp<3	svy, subpop(if target_dallas==1 & sitecode=="DA" & race_disp<3): tab rode_bicycle, col ci obs per format(%9.1f) 	svy, subpop(if rode_bicycle==1 & target_dallas==1 & sitecode=="DA" & race_disp<3): tab sex, col ci obs per format(%9.1f)	svy, subpop(if rode_bicycle==1 & target_dallas==1 & sitecode=="DA" & race_disp<3): tab race_disp, col ci obs per format(%9.1f) 	svy, subpop(if rode_bicycle==1 & target_dallas==1 & sitecode=="DA" & race_disp<3): tab age_cat, col ci obs per format(%9.1f) 	svy, subpop(if rode_bicycle==1 & target_dallas==1 & sitecode=="DA" & race_disp<3): tab year, col ci obs per format(%9.1f) 				//B. San Diego	tab rode_bicycle if year>1991 & sitecode=="SA" & target_cal==1  & race_disp<=3	svy, subpop(if year>1991 & target_cal==1 & sitecode=="SA" & race_disp<=3): tab rode_bicycle, col ci obs per format(%9.1f) 	svy, subpop(if rode_bicycle==1 & year>1991 & target_cal==1 & sitecode=="SA" & race_disp<=3): tab sex, col ci obs per format(%9.1f)	svy, subpop(if rode_bicycle==1 & year>1991 & target_cal==1 & sitecode=="SA" & race_disp<=3): tab race_disp, col ci obs per format(%9.1f) 	svy, subpop(if rode_bicycle==1 & year>1991 & target_cal==1 & sitecode=="SA" & race_disp<=3): tab age_cat, col ci obs per format(%9.1f) 	svy, subpop(if rode_bicycle==1 & year>1991 & target_cal==1 & sitecode=="SA" & race_disp<=3): tab year, col ci obs per format(%9.1f) 				//C. Florida counties: Intervention group	tab rode_bicycle if (sitecode=="MM" | sitecode=="FT") & target_florida==1 & race_disp<3	svy, subpop(if target_florida==1 & (sitecode=="MM" | sitecode=="FT") & race_disp<3): tab rode_bicycle, col ci obs per format(%9.1f) 	svy, subpop(if rode_bicycle==1 & target_florida==1 & (sitecode=="MM" | sitecode=="FT") & race_disp<3): tab sex, col ci obs per format(%9.1f)	svy, subpop(if rode_bicycle==1 & target_florida==1 & (sitecode=="MM" | sitecode=="FT") & race_disp<3): tab race_disp, col ci obs per format(%9.1f) 	svy, subpop(if rode_bicycle==1 & target_florida==1 & (sitecode=="MM" | sitecode=="FT") & race_disp<3): tab age_cat, col ci obs per format(%9.1f) 	svy, subpop(if rode_bicycle==1 & target_florida==1 & (sitecode=="MM" | sitecode=="FT") & race_disp<3): tab year, col ci obs per format(%9.1f) 						*========================================================================================**	TABLE 2: BEFORE-AFTER AND CHANGE IN DISPARITIES ANALYSES (ALSO SUPP. TABLES 2 & 3)	 |*========================================================================================*//A. Dallas	svy, subpop(if rode_bicycle==1 & target_dallas==1 & sitecode=="DA" & race_disp<3): glm helmet_s ib1995.year_disp_dallas##i.race_disp i.sex i.age_cat, link(log) family(bin) eform difficult cformat(%9.2f) 	margins rb1995.year_disp_dallas#r.race_disp, subpop(if rode_bicycle==1 & target_dallas==1 & sitecode=="DA" & race_disp<3) vce(uncond) cformat(%9.3f) 	margins rb1995.year_disp_dallas@i.race_disp, subpop(if rode_bicycle==1 & target_dallas==1 & sitecode=="DA" & race_disp<3) vce(uncond) cformat(%9.3f)//B. Florida counties	svy, subpop(if rode_bicycle==1 & target_florida==1 & (sitecode=="MM" | sitecode=="FT") & race_disp<3): glm helmet_s ib1995.year_disp_florida##i.race_disp i.sex i.age_cat, link(log) family(bin) eform difficult	margins rb1995.year_disp_florida#r.race_disp, subpop(if rode_bicycle==1 & target_florida==1 & (sitecode=="MM" | sitecode=="FT") & race_disp<3) vce(uncond) cformat(%9.3f)	margins rb1995.year_disp_florida@i.race_disp, subpop(if rode_bicycle==1 & target_florida==1 & (sitecode=="MM" | sitecode=="FT") & race_disp<3) vce(uncond) cformat(%9.3f)//C. San Diego	svy, subpop(if year>1991 & rode_bicycle==1 & target_california==1 & sitecode=="SA"): glm helmet_s ib1993.year_disp_california##i.race_disp i.sex i.age_cat, link(log) family(bin) eform difficult		margins rb1993.year_disp_california#r.race_disp, subpop(if year>1991 & rode_bicycle==1 & target_california==1 & sitecode=="SA") vce(uncond) cformat(%9.3f)	margins rb1993.year_disp_california@i.race_disp, subpop(if year>1991 & rode_bicycle==1 & target_california==1 & sitecode=="SA") vce(uncond) cformat(%9.3f)	*========================================================**	GRAPHS												 |*========================================================*//A. Dallas	svy, subpop(if rode_bicycle==1 & target_dallas==1 & sitecode=="DA" & race_disp<3): glm helmet_s i.year_disp_dallas##i.race_disp i.sex i.age_cat, link(log) family(bin) eform difficult	margins i.year_disp_dallas##i.race_disp, subpop(if rode_bicycle==1 & target_dallas==1 & sitecode=="DA" & race_disp<3) vce(uncond) 	marginsplot, name(dallas_timeseries, replace)	margins r.race_disp@i.year_disp_dallas, subpop(if rode_bicycle==1 & target_dallas==1 & sitecode=="DA" & race_disp<3) vce(uncond) 		marginsplot, yline(0) xdimension(year_disp_dallas) name(dallas_disparity_by_year, replace) 	margins rb1995.year_disp_dallas@i.race_disp, subpop(if rode_bicycle==1 & target_dallas==1 & sitecode=="DA" & race_disp<3) vce(uncond) 	marginsplot, yline(0) name(dallas_effect_by_year, replace)	margins rb1995.year_disp_dallas#r.race_disp, subpop(if rode_bicycle==1 & target_dallas==1 & sitecode=="DA" & race_disp<3) vce(uncond) 	marginsplot, yline(0) name(dallas_did_by_year, replace)			//B. Florida counties	svy, subpop(if rode_bicycle==1 & target_florida==1 & (sitecode=="MM" | sitecode=="FT") & race_disp<3): glm helmet_s i.year_disp_florida##i.race_disp i.sex i.age_cat, link(log) family(bin) eform difficult	margins i.year_disp_florida##i.race_disp, subpop(if rode_bicycle==1 & target_florida==1 & (sitecode=="MM" | sitecode=="FT") & race_disp<3) vce(uncond) 	marginsplot, name(florida_timeseries, replace)	margins r.race_disp@i.year_disp_florida, subpop(if rode_bicycle==1 & target_florida==1 & (sitecode=="MM" | sitecode=="FT") & race_disp<3) vce(uncond) 	marginsplot, yline(0) xdimension(year_disp_florida) name(florida_disparity_by_year, replace) 	margins rb1995.year_disp_florida@i.race_disp, subpop(if rode_bicycle==1 & target_florida==1 & (sitecode=="MM" | sitecode=="FT") & race_disp<3) vce(uncond) 	marginsplot, yline(0) name(florida_effect_by_year, replace)	margins rb1995.year_disp_florida#r.race_disp, subpop(if rode_bicycle==1 & target_florida==1 & (sitecode=="MM" | sitecode=="FT") & race_disp<3) vce(uncond) 	marginsplot, yline(0) name(florida_did_by_year, replace)		//C. San Diego	svy, subpop(if year>1991 & rode_bicycle==1 & target_california==1 & sitecode=="SA"): glm helmet_s i.year_disp_california##i.race_disp i.sex i.age_cat, link(log) family(bin) eform difficult	margins i.year_disp_california##i.race_disp, subpop(if year>1991 & rode_bicycle==1 & target_california==1 & sitecode=="SA") vce(uncond) 	marginsplot, name(sandiego_timeseries, replace)	margins r.race_disp@i.year_disp_california, subpop(if year>1991 & rode_bicycle==1 & target_california==1 & sitecode=="SA") vce(uncond)  	marginsplot, yline(0) xdimension(year_disp_california) name(sandiego_disparity_by_year, replace) 	margins rb1993.year_disp_california@i.race_disp, subpop(if year>1991 & rode_bicycle==1 & target_california==1 & sitecode=="SA") vce(uncond) 	marginsplot, yline(0) name(sandiego_effect_by_year, replace)	margins rb1993.year_disp_california#r.race_disp, subpop(if year>1991 & rode_bicycle==1 & target_california==1 & sitecode=="SA") vce(uncond) 	marginsplot, yline(0) name(sandiego_did_by_year, replace)		*========================================================================**	SUPP TABLE 1: UNAJDUSTED VERSION OF TABLE 1							 |*========================================================================*//A. Dallas	svy, subpop(if rode_bicycle==1 & target_dallas==1 & sitecode=="DA" & race_disp<3): glm helmet_s ib1995.year_disp_dallas##i.race_disp, link(log) family(bin) eform difficult cformat(%9.2f) 	margins rb1995.year_disp_dallas#r.race_disp, subpop(if rode_bicycle==1 & target_dallas==1 & sitecode=="DA" & race_disp<3) vce(uncond) cformat(%9.3f) 	margins rb1995.year_disp_dallas@i.race_disp, subpop(if rode_bicycle==1 & target_dallas==1 & sitecode=="DA" & race_disp<3) vce(uncond) cformat(%9.3f)		//B. Florida counties	svy, subpop(if rode_bicycle==1 & target_florida==1 & (sitecode=="MM" | sitecode=="FT") & race_disp<3): glm helmet_s ib1995.year_disp_florida##i.race_disp, link(log) family(bin) eform difficult	margins rb1995.year_disp_florida#r.race_disp, subpop(if rode_bicycle==1 & target_florida==1 & (sitecode=="MM" | sitecode=="FT") & race_disp<3) vce(uncond) cformat(%9.3f)	margins rb1995.year_disp_florida@i.race_disp, subpop(if rode_bicycle==1 & target_florida==1 & (sitecode=="MM" | sitecode=="FT") & race_disp<3) vce(uncond) cformat(%9.3f)	//C. San Diego	svy, subpop(if year>1991 & rode_bicycle==1 & target_california==1 & sitecode=="SA"): glm helmet_s ib1995.year_disp_california##i.race_disp, link(log) family(bin) eform difficult		margins rb1993.year_disp_california#r.race_disp, subpop(if year>1991 & rode_bicycle==1 & target_california==1 & sitecode=="SA") vce(uncond) cformat(%9.3f)	margins rb1993.year_disp_california@i.race_disp, subpop(if year>1991 & rode_bicycle==1 & target_california==1 & sitecode=="SA") vce(uncond) cformat(%9.3f)
