# Supplementary material for: Bicycle helmet laws and persistent racial and ethnic helmet use disparities among urban high school students: a repeated cross-sectional analysis
Source: Inj Epidemiol. 2016 Sep 5;3(1):21. doi: 10.1186/s40621-016-0086-3 (PMC5011068; doi:10.1186/s40621-016-0086-3)
Supplement: Additional file 2: — Appendix A: Supplemental Tables. (DOCX 100 kb) [file 40621_2016_86_MOESM2_ESM.docx]

**Appendix A: Supplemental Tables**

| **Supplemental Table S1.** Unadjusted increases in helmet use and disparities from before-to-after helmet law implementation. | | | | | | | | |
| --- | --- | --- | --- | --- | --- | --- | --- | --- |
|  | **Dallas** | | **San Diego** | | | | **Miami Dade & Broward Counties** | |
|  | *Mandate & Penalty* | | *Mandate Only* | | *Mandate & Penalty* | | *Mandate & Penalty* | |
|  | Δ% (CI) | p | Δ% (CI) | p | Δ% (CI) | p | Δ% (CI) | p |
| Before-to-After Increase in Helmet Use |  |  |  |  |  |  |  |  |
| White, Non-Latino | 21.1 (11.6, 30.7) | <0.001 | 18.3 (11.5, 25.0) | <0.001 | 24.6 (18.5, 30.8) | <0.001 | 10.1 (4.6, 15.6) | <0.001 |
| African American, Non-Latino | 7.9 (4.4, 11.3) | <0.001 | -0.1 (-6.2, 6.0) | 0.970 | 6.4 (-0.5, 13.2) | 0.069 | 6.5 (3.2, 9.8) | <0.001 |
| Latino | 6.2 (2.4, 10.1) | 0.002 | 4.1 (-1.9, 10.2) | 0.177 | 3.2 (-2.1, 8.5) | 0.233 | 4.4 (0.8, 7.9) | 0.015 |
| Asian | --- | --- | 11.9 (3.7, 20.0) | 0.004 | 12.4 (4.6, 20.2) | 0.002 | --- | --- |
| Increase in Disparity |  |  |  |  |  |  |  |  |
| White, Non-Latino | Ref. | Ref. | Ref. | Ref. | Ref. | Ref. | Ref. | Ref. |
| African American, Non-Latino | 13.3 (2.8, 23.8) | 0.013 | 18.4 (9.0, 27.8) | <0.001 | 18.3 (9.5, 27.1) | <0.001 | 3.6 (-2.5, 9.7) | 0.251 |
| Latino | 14.9 (4.8, 25.0) | 0.004 | 14.1 (5.4, 22.9) | 0.002 | 21.4 (13.0, 29.9) | <0.001 | 5.7 (0.0, 11.5) | 0.052 |
| Asian | --- | --- | 6.4 (3.8, 16.6) | 0.217 | 12.2 (3.1, 21.4) | 0.009 | --- | --- |
| Note: “---” denotes that the relevant subpopulation was not included because it was too small for stable estimates. | | | | | | | | |

| **Supplemental Table S2.** Adjusted differences in helmet use from the last pre-law year, stratified by jurisdiction and race/ethnicity. | | | | | | | | |
| --- | --- | --- | --- | --- | --- | --- | --- | --- |
|  | **White, Non-Latino** | | **African American, Non-Latino** | | **Latino** | | **Asian** | |
|  | Δ% (CI) | p | Δ% (CI) | p | Δ% (CI) | p | Δ% (CI) | p |
| Dallas |  |  |  |  |  |  |  |  |
| 1991 | 1.4 (-1.6, 4.4) | 0.356 | 0.1 (-1.4, 1.6) | 0.896 | 2.3 (-1.1, 5.7) | 0.178 | --- | --- |
| 1993 | 0.3 (-3.0, 3.6) | 0.861 | -0.3 (-1.8, 1.2) | 0.695 | 1.4 (-1.1, 3.9) | 0.272 | --- | --- |
| 1995 (last pre-law year) | Ref. | Ref. | Ref. | Ref. | Ref. | Ref. | --- | --- |
| 1997 | 6.7 (-0.2, 13.6) | 0.056 | 3.7 (1.3, 6.1) | 0.003 | 2.0 (-0.9, 4.9) | 0.185 | --- | --- |
| 1999 (first post-law year) | 20.1 (11.7, 28.5) | <0.001 | 8.2 (4.7, 11.7) | <0.001 | 6.2 (2.3, 10.0) | 0.002 | --- | --- |
| 2001/2003 | 13.0 (6.2, 19.9) | <0.001 | 6.8 (4.3, 9.3) | <0.001 | 4.8 (2.1, 7.5) | 0.001 | --- | --- |
| 2005/2007 | 13.0 (6.0, 20.0) | <0.001 | 4.0 (1.1, 6.8) | 0.006 | 3.7 (0.9, 6.5) | 0.010 | --- | --- |
| 2009/2011 | 6.6 (-1.1, 14.3) | 0.092 | 3.7 (0.7, 6.8) | 0.017 | 3.2 (0.5, 6.0) | 0.020 | --- | --- |
| San Diego |  |  |  |  |  |  |  |  |
| 1993 (last pre-law year) | Ref. | Ref. | Ref. | Ref. | Ref. | Ref. | Ref. | Ref. |
| 1995 (mandate only) | 18.3 (11.9, 24.8) | <0.001 | 0.3 (-5.8, 6.5) | 0.915 | 4.0 (-2.0, 9.9) | 0.188 | 11.9 (3.8, 20.0) | 0.004 |
| 1997 (mandate and penalty) | 24.4 (18.5, 30.3) | <0.001 | 6.3 (-0.5, 13.0) | 0.070 | 3.1 (-2.1, 8.3) | 0.242 | 12.8 (5.0, 20.5) | 0.001 |
| 1999 | 32.6 (24.5, 40.7) | <0.001 | 15.7 (6.7, 24.8) | 0.001 | 10.8 (5.0, 16.6) | <0.001 | 21.1 (12.1, 30.1) | <0.001 |
| 2001 | 36.6 (29.6, 43.7) | <0.001 | 17.5 (7.2, 27.7) | 0.001 | 9.2 (4.1, 14.2) | <0.001 | 17.0 (8.1, 25.9) | <0.001 |
| 2003/2005 | 34.8 (29.5, 40.1) | <0.001 | 7.4 (0.3, 14.5) | 0.041 | 7.1 (2.2, 12.0) | 0.004 | 15.1 (7.5, 22.8) | <0.001 |
| 2007/2009 | 32.7 (26.3, 39.0) | <0.001 | 2.4 (-3.3, 8.1) | 0.405 | 8.8 (4.0, 13.6) | <0.001 | 19.2 (11.2, 27.2) | <0.001 |
| 2011/2013 | 36.2 (30.4, 42.0) | <0.001 | 10.4 (2.3, 18.5) | 0.012 | 4.9 (0.6, 9.3) | 0.026 | 18.1 (9.3, 26.8) | <0.001 |
| Miami-Dade & Broward Co. |  |  |  |  |  |  |  |  |
| 1991 | -0.9 (-3.5, 1.7) | 0.495 | 0.7 (-0.7, 2.1) | 0.352 | -0.1 (-3.0, 2.7) | 0.923 | --- | --- |
| 1993 | -1.2 (-3.8, 1.4) | 0.359 | 4.1 (1.2, 7.0) | 0.006 | 0.3 (-2.9, 3.5) | 0.853 | --- | --- |
| 1995 (last pre-law year) | Ref. | Ref. | Ref. | Ref. | Ref. | Ref. | --- | --- |
| 1997 | 3.1 (-0.9, 7.1) | 0.132 | 1.7 (-0.1, 3.6) | 0.071 | 2.7 (-0.8, 6.2) | 0.129 | --- | --- |
| 1999 (first post-law year) | 10.2 (4.8, 15.7) | <0.001 | 6.1 (3.2, 9.0) | <0.001 | 4.3 (0.8, 7.9) | 0.016 | --- | --- |
| 2001 | 16.4 (11.0, 21.8) | <0.001 | 10.9 (6.1, 15.7) | <0.001 | 7.8 (3.0, 12.6) | 0.001 | --- | --- |
| 2003 | 14.1 (7.9, 20.3) | <0.001 | 4.8 (1.8, 7.9) | 0.002 | 6.1 (2.1, 10.1) | 0.003 | --- | --- |
| 2005 | 20.1 (12.5, 27.6) | <0.001 | 9.1 (5.4, 12.9) | <0.001 | 6.6 (3.1, 10.2) | <0.001 | --- | --- |
| 2007/2009 | 15.5 (7.5, 23.5) | <0.001 | 8.4 (5.2, 11.6) | <0.001 | 6.6 (3.4, 9.8) | <0.001 | --- | --- |
| 2011 | 13.3 (2.8, 7.9) | <0.001 | 5.3 (2.8, 7.9) | <0.001 | 9.3 (5.3, 13.3) | <0.001 | --- | --- |
| 2013 | 17.4 (9.3, 25.5) | <0.001 | 4.5 (1.7, 7.3) | 0.002 | 7.4 (3.4, 11.4) | <0.001 | --- | --- |
| Note: “---” denotes that the relevant subpopulation was not included because it was too small for stable estimates. | | | | | | | | |

| **Supplemental Table S3.** Adjusted increase in each subpopulation’s disparity in helmet use compared to the white subpopulation, stratified by jurisdiction. Comparisons are to the last pre-law year. | | | | | | |
| --- | --- | --- | --- | --- | --- | --- |
|  | **African American, Non-Latino** | | **Latino** | | **Asian** | |
|  | Δ% (CI) | p | Δ% (CI) | p | Δ% (CI) | p |
| Dallas |  |  |  |  |  |  |
| 1991 | 1.3 (-2.1, 4.7) | 0.456 | -0.9 (-5.2, 3.4) | 0.679 | --- | --- |
| 1993 | 0.6 (-3.1, 4.3) | 0.748 | -1.1 (-5.2, 3.0) | 0.592 | --- | --- |
| 1995 (last pre-law year) | Ref. | Ref. | Ref. | Ref. | --- | --- |
| 1997 | 3.0 (-3.5, 9.5) | 0.364 | 4.7 (2.6, 12.1) | 0.204 | --- | --- |
| 1999 (first post-law year) | 11.9 (2.3, 21.4) | 0.015 | 14.0 (4.7, 23.2) | 0.003 | --- | --- |
| 2001/2003 | 6.2 (-1.1, 13.5) | 0.094 | 8.3 (0.7, 15.8) | 0.032 | --- | --- |
| 2005/2007 | 9.0 (1.5, 16.6) | 0.019 | 9.3 (1.6, 16.9) | 0.018 | --- | --- |
| 2009/2011 | 2.9 (-5.4, 11.2) | 0.492 | 3.4 (4.5, 11.3) | 0.403 | --- | --- |
| San Diego |  |  |  |  |  |  |
| 1993 (last pre-law year) | Ref. | Ref. | Ref. | Ref. | Ref. | Ref. |
| 1995 (mandate only) | 18.0 (8.8, 27.3) | <0.001 | 14.4 (5.8, 22.9) | 0.001 | 6.4 (-3.9, 16.7) | 0.221 |
| 1997 (mandate and penalty) | 18.1 (9.3, 27.0) | <0.001 | 21.3 (13.0, 29.6) | <0.001 | 11.6 (2.5, 20.8) | 0.013 |
| 1999 | 16.9 (3.8, 29.9) | 0.011 | 21.8 (12.5, 31.1) | <0.001 | 11.5 (-1.2, 24.2) | 0.076 |
| 2001 | 19.2 (7.1, 31.2) | 0.002 | 27.5 (18.8, 36.1) | <0.001 | 19.6 (8.2, 31.1) | 0.001 |
| 2003/2005 | 27.4 (19.2, 35.7) | <0.001 | 27.7 (20.5, 35.0) | <0.001 | 19.7 (10.7, 28.7) | <0.001 |
| 2007/2009 | 30.3 (21.7, 38.9) | <0.001 | 23.8 (16.3, 31.3) | <0.001 | 13.5 (3.3, 23.6) | 0.010 |
| 2011/2013 | 25.8 (15.7, 35.9) | <0.001 | 31.3 (24.1, 38.5) | <0.001 | 18.2 (7.6, 28.7) | 0.001 |
| Miami-Dade & Broward Co. |  |  |  |  |  |  |
| 1991 | -1.6 (-4.5, 1.4) | 0.297 | -0.8 (-4.4, 2.8) | 0.681 | --- | --- |
| 1993 | -5.3 (-9.2, -1.4) | 0.008 | -1.5 (-5.4, 2.4) | 0.448 | --- | --- |
| 1995 (last pre-law year) | Ref. | Ref. | Ref. | Ref. | --- | --- |
| 1997 | 1.3 (-3.1, 5.8) | 0.558 | 0.4 (-4.8, 5.6) | 0.891 | --- | --- |
| 1999 (first post-law year) | 4.2 (-1.8, 10.1) | 0.169 | 5.9 (0.1, 11.7) | 0.045 | --- | --- |
| 2001 | 5.5 (-1.6, 12.5) | 0.127 | 8.6 (1.1, 16.2) | 0.025 | --- | --- |
| 2003 | 9.3 (2.3, 16.3) | 0.009 | 8.0 (1.3, 14.7) | 0.020 | --- | --- |
| 2005 | 10.9 (2.4, 19.4) | 0.012 | 13.4 (5.2, 21.6) | 0.001 | --- | --- |
| 2007/2009 | 7.1 (-1.2, 15.3) | 0.092 | 8.9 (0.5, 17.3) | 0.038 | --- | --- |
| 2011 | 8.0 (1.4, 14.6) | 0.017 | 4.0 (-2.4, 10.4) | 0.223 | --- | --- |
| 2013 | 12.9 (4.2, 21.7) | 0.004 | 10.0 (1.3, 18.8) | 0.025 | --- | --- |
| Note: “---” denotes that the relevant subpopulation was not included because it was too small for stable estimates. | | | | | | |
