# Supplementary material for: Bicycle helmet laws and persistent racial and ethnic helmet use disparities among urban high school students: a repeated cross-sectional analysis
Source: Inj Epidemiol. 2016 Sep 5;3(1):21. doi: 10.1186/s40621-016-0086-3 (PMC5011068; doi:10.1186/s40621-016-0086-3)
Supplement: Additional file 3: — Appendix B: Supplemental Figures. (DOC 333 kb) [file 40621_2016_86_MOESM3_ESM.doc]

**Appendix B: Supplemental Figures**

**Supplemental Figure S1.** Annual difference in helmet use from the last pre-law year.

*Note: Vertical reference lines correspond to the end of the pre-law period and start of the post-law period. Error bars represent 95% confidence intervals.*

**Supplemental Figure S2.** Annual disparity in helmet use compared to the white subpopulation.

*Note: Horizontal reference line corresponds to no disparity. Vertical reference lines correspond to the end of the pre-law period and start of the post-law period. Error bars represent 95% confidence intervals.*
